# Exploring the ER channel protein Sec61: recent advances in pathophysiological significance and novel pharmacological inhibitors

**DOI:** 10.3389/fphar.2025.1580086

**Published:** 2025-06-04

**Authors:** Jingxin Xin, Keling Yin, Shimeng Li, Peiyuan Gu, Shanshan Shao

**Affiliations:** ^1^ Department of Endocrinology, Shandong Provincial Hospital, Shandong University, Key Laboratory of Endocrine Glucose & Lipids Metabolism and Brain Aging, Ministry of Education, Jinan, Shandong, China; ^2^ Department of Endocrinology, Shandong Provincial Hospital Affiliated to Shandong First Medical University, Jinan, Shandong, China; ^3^ Shandong Key Laboratory of Endocrine Metabolism and Aging, Jinan, Shandong, China; ^4^ Shandong Institute of Endocrine and Metabolic Diseases, Jinan, Shandong, China; ^5^ “Chuangxin China” Innovation Base of stem cell and Gene Therapy for endocrine Metabolic diseases, Jinan, Shandong, China; ^6^ Shandong Engineering Laboratory of Prevention and Control for Endocrine and Metabolic Diseases, Jinan, Shandong, China

**Keywords:** Sec61, endoplasmic reticulum, genetic disease, tumor, inhibitor

## Abstract

The Sec61 complex, which is located on the membrane of the mammalian endoplasmic reticulum (ER), serves as a pivotal component of protein transport channels. It plays a central role in the transport of nascent peptides and precursor peptides to the ER. This process includes the directed movement of precursor peptides to the ER membrane and the opening of the Sec61 transduction channel for translocation. The Sec61 channel not only plays a key role in transporting peptides into cells but also acts as a passive ER Ca^2+^ leak channel. In addition, the mutation, amplification and overexpression of Sec genes are closely related to the development of various genetic diseases and cancers. Over the past few decades, studies have elucidated the function of the Sec61 protein in the pathogenesis of diseases such as cancer, and Sec61 inhibitors have been developed for their treatment. This review describes the structure of Sec61 and its function in transporting ER transmembrane proteins and further summarizes the role of this gene in disease and recent advancements in Sec61 inhibitors. This study provides novel insights into the involvement of Sec61 in disease etiology and lays the groundwork for future treatment modalities targeting this pivotal protein complex.

## 1 Introduction

**FIGURE 1:**
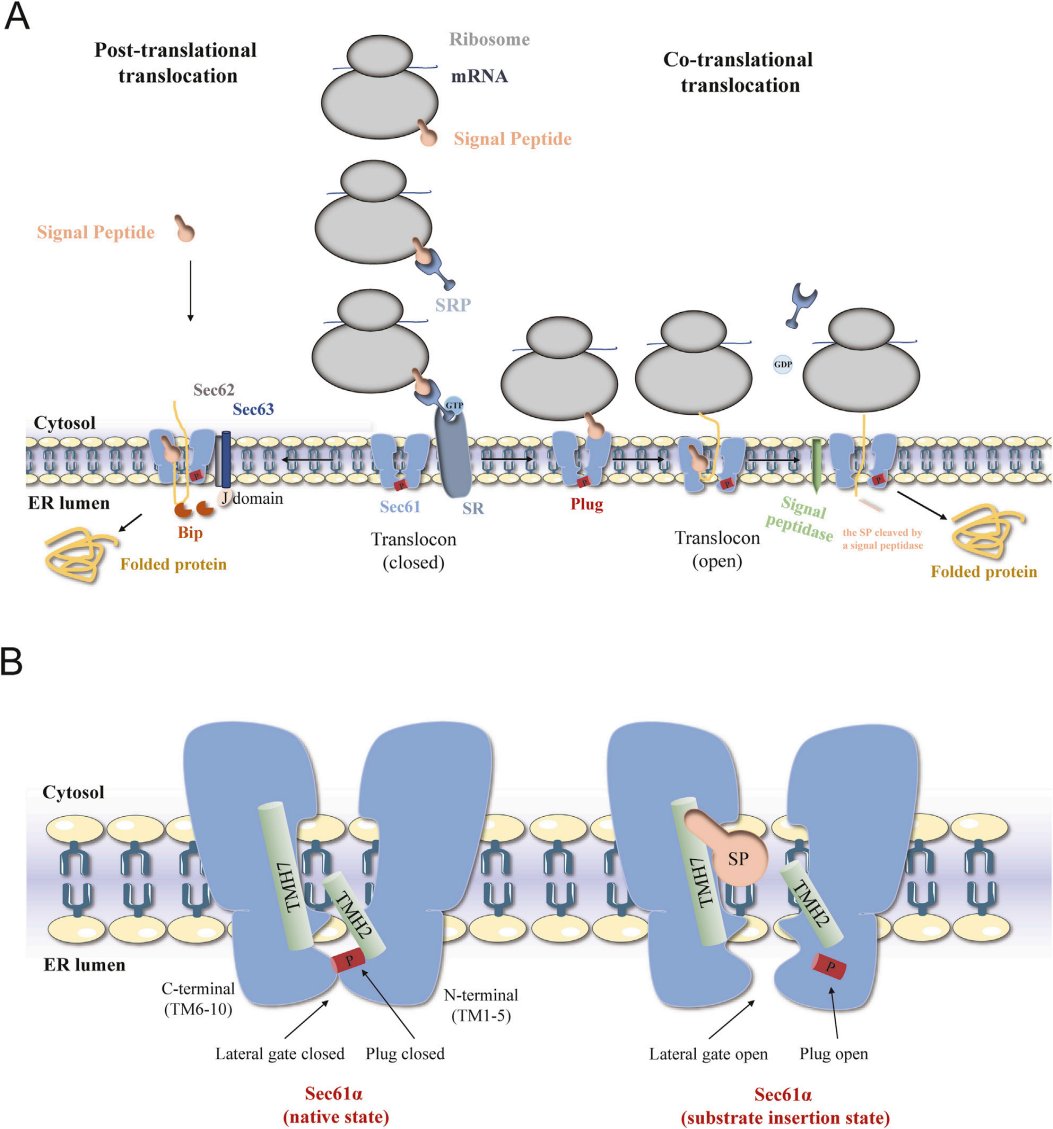
The schematic diagram of Sec61 translocation process and translocation conformations. **(A)** Schematic diagrams of the mechanisms of posttranslational transport (left) and cotranslational transport (right) of proteins mediated by the Sec61 complex in eukaryotic cells. **(B)** Overview of different structural states of SEC61α translocon. Conformation of Sec61α in the Sec61 complex in the resting state (left) and during translocation (right).

In eukaryotic cells, the folding and assembly of most secreted proteins and transmembrane proteins are completed in the endoplasmic reticulum (ER). The entry of nascent peptides into and across the ER membrane is a highly conserved process that requires the participation of various translocation proteins, including the ER channel protein Sec61 (Lang et al., 2019). Depending on the translocation of precursor proteins during or after ribosome synthesis, their transport across the endoplasmic reticulum membrane can be classified into two modes: cotranslational transport (Rapoport et al., 1996; Matlack et al., 1998) and posttranslational transport (Schlenstedt and Zimmermann, 1987; Kutay et al., 1995) (Figure 1A). During cotranslational transport, the signal peptide (SP) emerging from the ribosome is recognized by the cytosolic signal recognition particle (SRP), which causes translation to slow down. Then, SRP interacts with the SRP receptor (SR) located in the ER membrane to form a ribosome - nascent chain complex, which is guided to the Sec61 complex on the ER membrane (Rapoport et al., 2017). Translation resumes, and the nascent polypeptide chain enters the ER lumen through the Sec61 channel. The SP is cleaved by a signal peptidase located in the ER lumen, and the polypeptide chain undergoes folding and modification within the ER. Meanwhile, guanosine triphosphate (GTP) hydrolysis enables SRP and SR to return to their original state, preparing for the next round of cotranslational translocation (Haßdenteufel et al., 2019). In the posttranslational transport pathway, secretory proteins with fewer hydrophobic signal sequences bypass the SRP and complete translation on cytoplasmic free ribosomes (Schorr et al., 2020; Schlenstedt et al., 1990). Subsequently, signal proteins are guided into the ER by the Sec61 channel and the Sec62/Sec63 complex, after which they undergo folding and covalent modification (Deshaies et al., 1991; Kreil, 1981). In this process, as an endoplasmic reticulum lumen chaperone protein, the immunoglobulin heavy chain binding protein BiP/GRP78 acts as a “one-way valve” to ensure the unidirectional transport of signal proteins to ER tubes through the Sec61 channel (Dudek et al., 2009; Alder et al., 2005). This paper reviews the function of the Sec61 protein in cotranslational transport and further focuses on recent studies that provided the first insights into the functional role and therapeutic relevance of Sec61 in human diseases.

## 2 Structure and function of Sec61

The eukaryotic Sec61 complex is composed of three subunits, Sec61α, Sec61β and Sec61γ (referred to as Sec61p, Sbh1 and Sss1, respectively, in *Saccharomyces cerevisiae*) (Van den Berg et al., 2004). The Sec61α and Sec61γ subunit sequences are highly conserved and are critical for protein transport and cell viability, while the Sec61β subunit has low sequence homology and a limited role in the function of the channel (Tsukazaki et al., 2008). Sec61α, the largest subunit, contains ten transmembrane domains (TMDs). Transmembrane helices (TMs) 1-5 and TMs 6–10 form the central pore plug - like domains. These TM domains collectively create a clam - shaped pore with a central ring, facilitating protein translocation. On the luminal side, a short helix (TM2a) forms a displaceable plug domain that can seal the pore (Heinrich et al., 2000). Initial photocrosslinking experiments showed that the Sec61α subunit was surrounded by the peptide chain as it passed through the channel (Mothes et al., 1994). Sec61β and Sec61γ are single transmembrane proteins that belong to the tail-anchored protein family. The Sec61 channel exhibits a characteristic clamshell-like topology, with the central pore opening into the lipid phase through a lateral gate formed between TM2 and TM7 (Li et al., 2016). The channel in the Sec61 complex has a larger opening at the side gate to facilitate the passage of signal sequences in the form of α-helixes. Currently, the cryo-electron microscopic structure of the Sec61 complex has been used to determine several functional conformations (Bai et al., 2020). A comparison of the open, shifted, and idle conformations of Sec61α suggested that changes in the lateral gate conformation may be due to an unstable hydrogen bond network of pore ring residues located on TM2, TM5, TM7, and TM10. According to the two cryo-EM images of the mammalian ribosome-bound Sec61 complex (cotranslational mode), a hydrophobic signal peptide was observed to occupy the space between TM2 and TM7 and was eventually inserted into the lipid bilayer with a helical structure (Weng et al., 2021). To analyze the conformational changes in the Sec61 channel, Sun et al. conducted molecular dynamics (MD) simulations of the mammalian Sec61 channel and revealed that after the signal peptide chain enters the lipid bilayer, the side gate can quickly return to the partially closed state, and the conformational dynamics of the side gate, the pore loop and the plug domain are interrelated (Sun et al., 2019). Molecular docking and experimental results indicated that the hydrophobic core of the SRP substrate-dependent signal anchor was more inclined to occupy the space between the C-terminus of TM2 and the N-terminus of TM7 than was the hydrophobic core of the SRP substrate-independent signal peptide (Salmaso and Moro, 2018; Bhadra and Helms, 2021). These results indicate that the translocation process is dependent on the interaction of the targeting sequence with the side gate (Figure 1B).

The core Sec61 complex is highly dynamic. It can interact with a variety of molecular machines and enzymes to form multiple unique subcomplexes, each with distinct client proteins. These subcomplexes include key players in canonical translocation like the translocon - associated protein complex (TRAP), translocating - chain - associating membrane protein (TRAM), oligosaccharyltransferase (OST), and ribosome (Itskanov and Park, 2023; Pfeffer et al., 2014). There are also recently identified ones such as the EMC (ER membrane protein complex), GEL (Guided entry of tail-anchored proteins and EMC-Like) complex, PAT (protein associated with translocon), and BOS (Back of Sec61) complexes (Page et al., 2024). Studies show that in the biogenesis of tail - anchored (TA) proteins via either the GET or EMC pathway, the hydrophobicity of the transmembrane domain (TMD) is the key determinant, which is especially important for the insertion of multi - pass membrane proteins, including GPCRs. Notably, the EMC complex can substitute for Sec61 in inserting type III membrane proteins (Page et al., 2024). By associating with these complexes, the Sec61 complex is activated, a driving force for translocation is generated, and polypeptide translocation is coupled with other processes such as translation, post - translational modifications, and protein folding. Thus, the Sec61 complex has evolved to be regulated in a substrate - and partner complex - dependent manner to ensure the efficiency and accuracy of protein transport and insertion.

The Sec61 complex on the ER membrane serves as the primary entry channel for nascent polypeptides and possesses three protein transport functions: (1) by forming a protein transport channel; (2) by recognizing a functional signal sequence; and (3) by serving as the main ribosome receptor. During the ER targeting and transport processes, Sec61 enters the open state and interacts with a variety of other protein complexes on the cytoplasmic surface and on the ER membrane. This process is promoted not only by its substrate, amino terminus or transmembrane helical region of the signal peptide (SP) but also by translation transport ribosomes, TRAP and Sec62/Sec63 complex (Kalies et al., 2008; Almagro Armenteros et al., 2019; Voorhees and Hegde, 2016; Pfeffer et al., 2017; Itskanov et al., 2021). If proteins are not correctly folded in the ER, they trigger the unfolded protein response (UPR), or they are transported back to the proteasome for ER-associated protein degradation (ERAD) (Pilla et al., 2017; Moon et al., 2018; Fregno and Molinari, 2019). In addition, the Sec61 channel is considered a passive Ca^2+^ leak channel on the ER membrane that allows Ca^2+^ efflux from the ER in all nucleated cells (Van Coppenolle et al., 2004). In the case of severe and prolonged protein misfolding and aggregation, BiP isolates misfolded and aggregated peptides, leading to sustained Ca^2+^ leakage through the open Sec61 channel. Research indicates that various human genetic diseases and tumor diseases are caused by Sec61 point mutations and are associated with Sec61 channel gating dysfunction, which will be reviewed in detail later (Lang et al., 2017; Sicking et al., 2021; Linxweiler et al., 2017).

## 3 Sec61 proteins and diseases

In recent years, mutations and overexpression of Sec61 have been linked to numerous human diseases.

### 3.1 SEC61 mutation and genetic diseases

Diabetes mellitus (DM) is a metabolic disorder characterized by elevated blood glucose levels due to either an inadequate response to insulin or insufficient insulin production. According to a recent study on diabetes from 1999 to 2022, the global number of adults with diabetes reached 828 million in 2022, over four times the 1990 number. In terms of countries, in 2022, of the 828 million adults with diabetes globally, 212 million were in India (over a quarter), 148 million in China, 42 million in the US, 36 million in Pakistan, 25 million in Indonesia, and 22 million in Brazil (NCD Risk Factor Collaboration NCD-RisC, 2024). Proinsulin enters the ER through the Sec61-mediated cotranslational transport pathway (Liu et al., 2014). A mutation at the Y344H site of the SEC61A1 gene (where histidine at position 344 is replaced by tyrosine) can cause ER stress in the pancreatic islets of C57BL/6 mice. This leads to the apoptosis of pancreatic islet β-cells, results in insufficient insulin secretion, and eventually causes diabetes (Lloyd et al., 2010). However, when screening substrates, researchers have shown that compared with normal mice, heterozygous SEC61α^+/Y344^ and homozygous SEC61α^Y344H/Y344H^ mice exhibit reduced expression levels of the ERj3 protein in pancreatic and hepatic tissues (Schorr et al., 2020). In HeLa cells, when wild-type SEC61α is replaced with the corresponding mutant SEC61α^Y344H^, ER calcium leakage increases and is no longer affected by the BiP concentration (Schäuble et al., 2012). This finding suggested that BiP normally mediates the closure of the Sec61 channel to limit Ca^2+^ leakage from the ER. Therefore, in different tissues of adult mice, ERj3 was confirmed to enter the mammalian ER by interacting with Sec61α TM7 in a BiP-dependent manner. Notably, many studies have confirmed that mutations and deletions of other resident ER proteins can also affect the biosynthesis of proinsulin and insulin and contribute to the development of DM, such as the deletion of the Hsp40-type accessory chaperones ERj4 and ERj5 of BiP or mutations of the BiP-interacting proteins proline-rich receptor-like protein kinase (PERK) and TRAP (Fritz et al., 2014; Dong et al., 2008; Harding et al., 2001; Huang et al., 2021).

Autosomal-dominant tubulo-interstitial kidney disease (ADTKD) is a monogenic disease characterized by renal tubular damage and interstitial fibrosis without glomerular damage and can lead to chronic progressive loss of renal function, which inevitably leads to end-stage renal disease (Devuyst et al., 2019). A SEC61A1 heterozygous mutation was detected in an ADTKD family, as were missense mutations at V67G (located in the plug domain) and T185A (located near the TM 5-hole loop). Both of these mutations affect important functional and conserved residues in Sec61, thereby causing renal tubule atrophy. These data were confirmed in a study of zebrafish embryos; replacement of either of these two variants affects the development of the anterior kidney and results in a coiling defect in the anterior tubules, which is consistent with the renal tubular atrophy observed in patients (Bolar et al., 2016). Moreover, in HEK293 cells, these two mutants caused the Sec61 protein to aggregate into clumps in the ER and appear in the Golgi apparatus. This abnormal protein may be mislocated to the endoplasmic reticulum-Golgi intermediate compartment (ERGIC) and subjected to ER-related protein degradation. Therefore, Sec61α is essential for the development and maintenance of tubular tissues in the nephron.

Common variable immune deficiency (CVID) is a group of diseases of different origins that are usually characterized by impaired B-cell differentiation and function, resulting in low levels of immunoglobulin production and leading to respiratory infections in patients with repeated severe infections of multiple systems (Schubert et al., 2018). SEC61A1 deficiency is one underlying cause of CVID and is attributed to the missense mutation Sec61α V85D (in TM2) and the premature stop nonsense mutation E381* (in TM8). In addition, the overexpression of Sec61α V85D in HeLa cells not only affected the cotranslational transport of proteins but also greatly increased Ca^2+^ leakage from the ER, precipitating ER stress and irreversible UPR. Despite normal peripheral B and T-cell subsets in the two mutations, the production of SEC61α V85D selectively impaired the survival of cells in the plasma cell lineage because SEC61A1 is a target gene of XBP1s during plasma cell differentiation. B-cell lines transformed with EBV harboring the SEC61A1 mutation secreted less immunoglobulin. Currently, patients are responding well to immunoglobulin replacement therapy.

Severe congenital neutropenia (SCN) includes a group of genetically heterogeneous congenital immune deficiencies that are characterized by the arrest of granulocyte production and differentiation at the promyelocytic stage. Starting in early childhood, the absolute number of circulating mature neutrophils is low, predisposing SCN patients to life-threatening and recurrent infections (Skokowa et al., 2017). In patients with autosomal dominant severe congenital neutropenia (ADSCN), the identified heterozygous SEC61A1 mutations included two missense mutations, which led to amino acid substitutions, namely, V67G (in the plug domain) and Q92R (in TM2) (Van Nieuwenhove et al., 2020). Interestingly, neutropenia was also observed in patients with ADTKD harboring the SEC61α V67G mutation. Similarly, patients with the Q92R mutation not only had features of the SCN but also had B-cell maturation defects. In contrast, the kidney morphology of patients with the Q92R mutation was normal, and renal function also remained normal. These two mutations were both observed to cause reduced cellular Sec61 levels due to protein instability and dysregulation of calcium homeostasis. In addition, in myeloid leukemia HL-60 cells, after replacement of wild-type Sec61α with the Q92R mutant, calcium leakage from the ER increased, and differentiation into CD11b^+^ and CD16^+^ cells decreased, suggesting that the UPR was dysregulated. This finding was confirmed by *ex vivo* single-cell analysis (Van Nieuwenhove et al., 2020) (Table 1). In addition to mutations at the SEC61A1 locus causing SCN, the SRP also plays a critical role in neutrophil development. It has been found that human genetic defects in SRP19, SRPRA, and SRP54 cause severe congenital neutropenia (Linder et al., 2023; Bellanné-Chantelot et al., 2018).

**TABLE 1:** SEC61 mutation and genetic diseases.

Sec61A mutation site	Mutation position	Primary disease	Pathogenic mechanism	References
Y344H	TM7	DM	Pancreatic islets and islet β-cell apoptosis, insufficient insulin secretion	Schorr et al. (2020), Lloyd et al. (2010), Schäuble et al. (2012)
V67G	Located in the plug-like domain between TM1 and TM2	ADTKD, SCN	Renal tubular atrophy, neutropenia	Bolar et al. (2016)
T185A	TM5	ADTKD	Renal tubular atrophy	Bolar et al. (2016)
V85D	In TM2	CVID	Normal peripheral B and T-cell subsets, reduced plasma cells and reduced immunoglobulin	Schubert et al. (2018)
E381*	In TM8	CVID	Reduced immunoglobulin	Schubert et al. (2018)
Q92R	In TM2	SCN, CVID	Other leukopenia, B-cell maturation defect, and the morphology and function of kidney were normal	Van Nieuwenhove et al. (2020)

### 3.2 Sec61 and cancer

**FIGURE 2:**
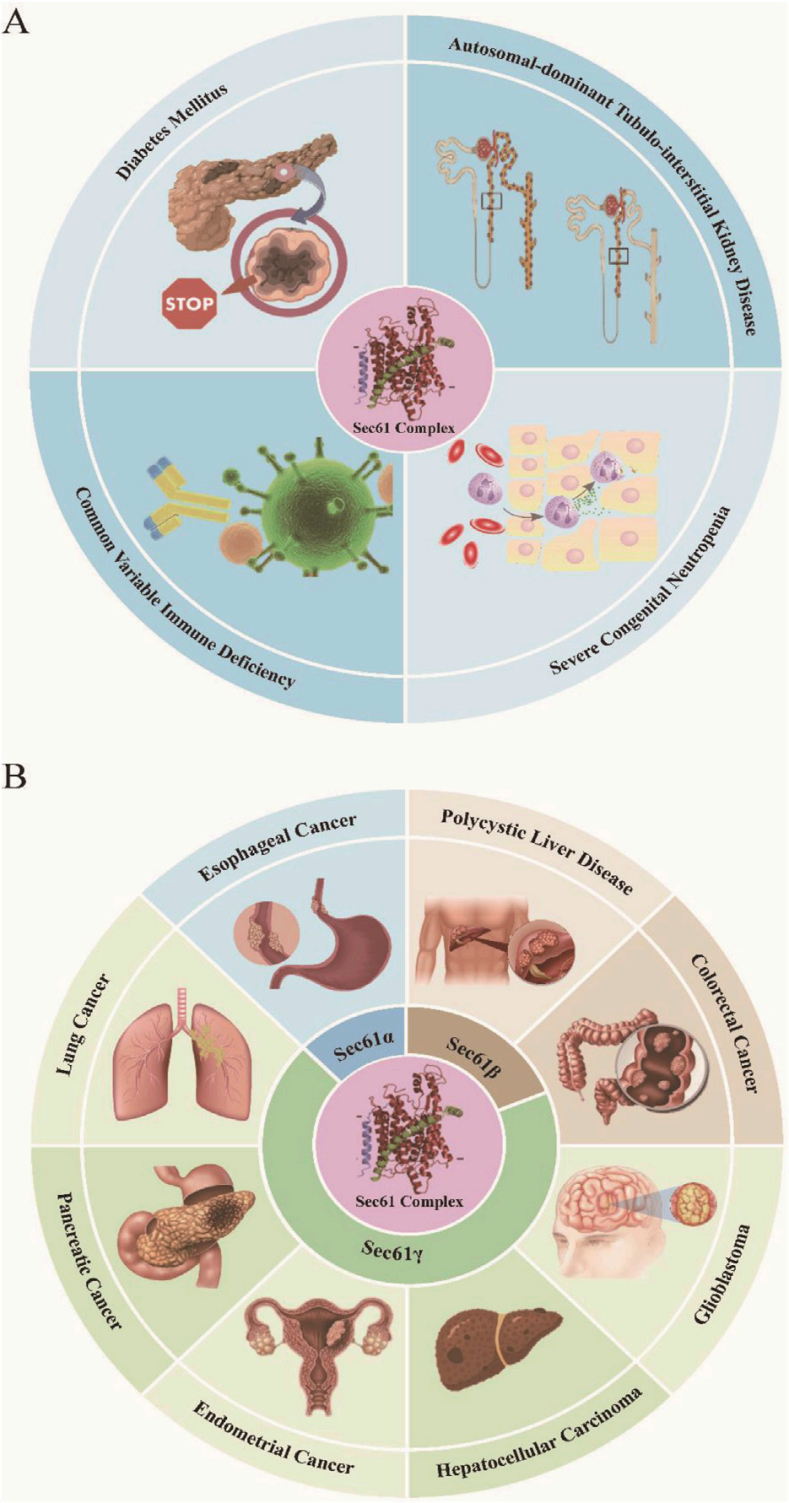
Mutations and overexpression of SEC61 gene in numerous human genetic disease **(A)** and cancer **(B)**.

There are many reports on the upregulation of SEC61 gene expression in malignant tumors. Sec61α is highly expressed in esophageal cancer, but this expression is not related to patient prognosis (Bachmann et al., 2019). Fan and his colleagues reported that the expression of Sec61β was significantly increased in colorectal cancer (CRC) patients. The Sec61β autoantibody level in the plasma of patients was also significantly greater than that in the plasma of healthy controls, suggesting that the Sec61β autoantibody can be used as a new serum marker of CRC, especially in the early stage (Fan et al., 2011). In addition, according to whole-exome sequencing of polycystic liver disease (PCLD), in addition to the two most common genes, PRKCHS and SEC63, deletion of the Sec61β gene results in severely reduced expression of polycystin-1, which is encoded by the PKD1 gene. It is also a pathogenic inducer of polycystic liver disease (Besse et al., 2017). Lu et al. used quantitative polymerase chain reaction (PCR) to measure gene-level changes in 43 human glioblastoma patients and reported that 77% of patients had increased expression of SEC61G, while the expression of the genes encoding SEC61A1 and SEC61B did not differ from that of healthy individuals (Lu et al., 2009). On the basis of the statistical analysis of sequencing data from the Cancer Genome Atlas (TCGA) and the Chinese Glioma Genome Atlas (CGGA) cohorts, researchers have shown that high SEC61G expression is significantly associated with poor prognosis in glioblastoma patients. SEC61G may be used as a new prognostic marker for predicting the survival and treatment response of patients with glioblastoma (Liu et al., 2019). In addition, SEC61γ is a prognostic marker for hepatocellular carcinoma (HCC). SEC61G expression is significantly upregulated in HCC and is associated with patient survival (Gao et al., 2020). By analyzing the COSMIC database, six SEC61γ mutations were found in highly conserved residues in eukaryotes. The R24I mutation was found in a patient with colorectal cancer, the K27E and I64T mutations were found in endometrial cancer patients, the A39V mutation was found in pancreatic cancer patients, and the L56F and H58R mutations were found in lung cancer patients (Witham et al., 2021). The cancer-associated SEC61γ mutants are proposed to alter ion transport across the channel, such as GSH, Mn^2+^ and Ca^2+^, yet they do not impair the ability of this protein complex to transfer secreted proteins to the ER (Witham et al., 2021) (Figure 2).

## 4 Small molecule inhibitors of Sec61

Studies have shown that selective inhibitors of secreted proteins can prevent cotranslational translocations by directly targeting Sec61 (Garrison et al., 2005). The correct translocation of proteins is critical for the normal functioning of cells. Since many inhibitors share the binding region of the Sec61α subunit (Paatero et al., 2016; Zong et al., 2019; Baron et al., 2016), Sec61α shows great potential as a molecular target for the treatment of various conditions, such as cancer, immune disorders, and viral infections (Pauwels et al., 2021a). These Sec61-dependent inhibitors are classified into substrate-selective inhibitors, such as HUN-7293, CAM741, Cotransin, and CADA, and broad-spectrum inhibitors, including Mycolactone, Exotoxin A, Apratoxin A, Coibamide A, Ipomoeassin F, Decatransin and Eeyarestatins (Table 2). These inhibitors have been proven to block the translocation of signal proteins and inhibit the leakage of Ca^2+^ through the Sec61 channel (Linxweiler et al., 2017; Haßdenteufel et al., 2018). In fact, SEC61a mutants that are resistant to one inhibitor usually also develop resistance to other inhibitors. Nevertheless, different inhibitors exhibit different specificities for transporters in different species and block the translocation of different types of substrates, which provides the possibility of developing therapeutically effective selective transporter modulators.

**TABLE 2:** Chemical information for the Sec61 inhibitors.

Inhibitor	Molecular formula	PubChem CID	Substrate selectivity	Chemical structures
HUN-7293	C_52_H_82_N_8_O_8_	10931051	VCAM-1,ICAM-1,E-selectin	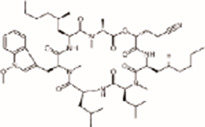
CAM741	C_56_H_91_N_7_O_11_	102353267	VCAM-1,VEGF	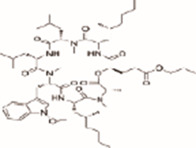
Cotransin	C_42_H_68_N_6_O_8_	25068231	Angiotensinogen,VCAM-1,p-selectin,β-lactamase,CRF1,ETBR,AQP2,TNFα,HER3	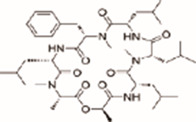
CADA	C_31_H_39_N_3_O_4_S_2_	466371	huCD4, SORT, DNAJC3, PTK7, ERLEC1, 4-1BB	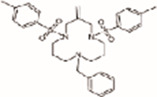
Mycolactone	C_44_H_70_O_9_	5282079	Broad-spectrum	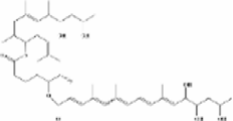
Exotoxin A	C_25_H_25_N_9_O_2_	135345207	Broad-spectrum	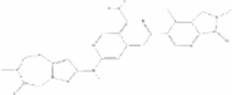
Apratoxin A	C_45_H_69_N_5_O_8_S	6326668	Broad-spectrum	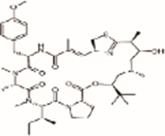
Coibamide A	C_65_H_110_N_10_O_16_	24881184	Broad-spectrum	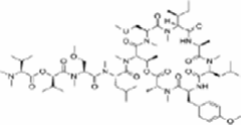
Ipomoeassin F	C_44_H_62_O_15_	25258999	Broad-spectrum	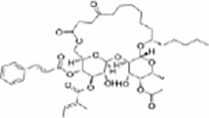
Decatransin	C_63_H_109_N_9_O_12_	166642447	Broad-spectrum	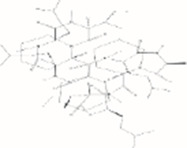
Eeyaresatin	C_27_H_25_Cl_2_N_7_O_7_	5003929	Broad-spectrum	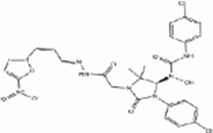

### 4.1 SEC61 substrate-selective inhibitors

#### 4.1.1 HUN-7293, CAM741 and cotransin

Cell adhesion molecules play an important role in the immune response by regulating the migration of leukocytes at sites of inflammation and interactions between cells. The first substrate-selective inhibitor discovered was HUN-7293, which inhibits the expression of three endothelial cell adhesion molecules: intercellular adhesion molecule 1 (ICAM-1), vascular cell adhesion molecule (VCAM-1), and E-selectin (Foster et al., 1994). CAM741 is an analog of Hun-7293 that is thought to interfere with the correct positioning of the VCAM - one signal peptide at the lateral gate upon its insertion into the translocase after being released from the SRP. Another substrate of CAM741 is the vascular endothelial growth factor (VEGF) (Harant et al., 2007). It can also prevent signal proteins from entering the lumen of the ER, indicating that this compound inhibits the signal peptide-dependent gating of the Sec61 channel (Westendorf et al., 2011). Another HUN-7293 analog named cotransin, inhibits the co-translational translocation of specific substrate proteins across the ER membrane. Numerous substrates have been identified for cotransin, including angiotensinogen, VCAM-1, p-selectin, β-lactamase, corticotropin-releasing factor 1 (CRF1), endothelin B receptor (ET_B_R), aquaporin 2 (AQP2), TNFα, and others (Garrison et al., 2005; Westendorf et al., 2011; Mackinnon et al., 2014; Klein et al., 2015; Maifeld et al., 2011). Considering the pivotal roles of VCAM-1, ICAM-1, and TNFα in cellular immune responses, HUN7293 and its related molecules CAM741 and cotransin are potentially promising as immunosuppressive agents (Maifeld et al., 2011). Additionally, cotransin has been found to target the oncoprotein human epidermal growth factor receptor 3 (HER3), which implies that cotransin may also possess anticancer activity (Ruiz-Saenz et al., 2015). Researchers have demonstrated the importance of Sec61 in supporting the replication of influenza A virus (IAV), human immunodeficiency virus (HIV), and dengue virus by using Cotransin to block the Sec61 channel, suggesting that inhibiting protein translocation across the ER is a potential antiviral strategy (Shah et al., 2018; Heaton et al., 2016).

#### 4.1.2 Cyclotriazadisulfonamide

Cyclotriazadisulfonamide (CADA) is a synthetic small-molecule translocation inhibitor and the first such inhibitor found to directly bind to signal peptides. To date, six substrates of CADA have been identified (Claeys et al., 2021; Pauwels et al., 2021b): huCD4, SORT, DNAJC3, PTK7, ERLEC1, and 4-1BB (CD137), highlighting its substrate selectivity. By acting in a signal peptide-dependent manner, CADA inhibits the co-translational translocation of the type I integral membrane protein human CD4 (huCD4) across the endoplasmic reticulum (Vermeire et al., 2002). This mechanism allows CADA to specifically target proteins with signal peptides, thereby affecting their intracellular transport and localization. In addition, CADA can also significantly downregulate the expression of huCD4 on the surface of various cells, such as monocytes, T cells, and other lymphocytes (Pauwels et al., 2021b; Vermeire et al., 2002; Vermeire and Schols, 2003). As huCD4 is the primary receptor for HIV entry into host cells, CADA’s presence makes it difficult for HIV to bind to huCD4, thus inhibiting viral infection and replication and demonstrating significant antiviral effects (Vermeire et al., 2004). CADA also suppresses the expression of other membrane proteins related to viral entry, such as SORT, further enhancing its antiviral activity (Pauwels et al., 2021b). Moreover, CADA exerts immunosuppressive effects by inhibiting the secretion of multiple cytokines and reducing the levels of CD25, phosphorylated STAT5, and CTPS-1 (Claeys et al., 2021). Its impact is particularly pronounced in CD8^+^ T cell subsets, where it inhibits cell-mediated lympholysis. Notably, this effect is associated with CADA-induced upregulation of CD137, as CADA upregulates CD137 to suppress immune cell activation and function, thereby achieving an immunosuppressive effect (Claeys et al., 2021). Furthermore, CADA is associated with a reduction in progranulin - induced breast cancer stem cell proliferation, indicating its potential as an anticancer agent (Berger et al., 2021).

### 4.2 Sec61 broad-spectrum inhibitors

#### 4.2.1 Mycolactone

Mycolactone inhibits the co-translational translocation of proteins into the ER through a mechanism that does not compromise the ER’s structural integrity (Hall et al., 2014). Secreted by the pathogen *Mycobacterium* ulcerans, this diffusible, lipid-like exotoxin forms a stable complex with Sec61a (Baron et al., 2016; Yotsu et al., 2018; Demangel and High, 2018). At nanomolar concentrations, it can block the co-translational transport of secreted proteins, such as various inflammatory mediators and cytokines, and inhibit the co-translational translocation of Sec61-dependent secreted proteins (McKenna et al., 2016). Mycolactone inhibits the translocation stage following ribosome contact with the translocon, affecting signal peptide interactions. This potent and stable inhibitor provides an opportunity to observe transporters in an inhibited state. Mycolactone can serve as a therapeutic drug with minimal side effects for multiple myeloma (MM), and it can effectively reduce the resistance of MM to proteasome inhibitors and immunomodulatory drugs. It induces ER stress *in vitro*, leading to the death of MM cell lines. In immunodeficient mice transplanted with MM cells, both primary and relapsed MM tumors were killed, and the growth of MM xenografts was delayed (Domenger et al., 2022).

#### 4.2.2 Exotoxin A

Exotoxin A of *Pseudomonas aeruginosa* is a cytotoxic ADP-ribosyltransferase. It enters the cytoplasm of eukaryotic cells via endocytosis and retrograde transport. Moreover, it inhibits the retrograde export of immunogenic peptides from the endoplasmic reticulum to the cytoplasm (Zehner et al., 2015). Exotoxin A also competes with the cytoplasmic protein calmodulin (CaM) for binding to the N-terminus of Sec61α, thereby closing the Sec61 channel, preventing Ca^2+^ leakage, and terminating the cotranslational and posttranslational transport of signal proteins (Schäuble et al., 2014). Currently, the effectiveness of exotoxin A in antitumor applications has been validated in clinical studies. However, it can also damage the immune system of infected patients and may cause pneumonia or sepsis. At present, recombinant toxins with improved immunogenicity and reduced toxicity can be constructed through genetic engineering techniques to enhance their efficacy and reduce adverse effects (Burkhardt et al., 2019).

#### 4.2.3 Apratoxin A and Coibamide A

Apratoxin A and coibamide A are natural secondary metabolites isolated from marine cyanobacteria (Tranter et al., 2020). They are produced by a nonribosomal peptide synthetase. Apratoxin A was found to be a cytotoxic antitumor drug capable of inhibiting the growth of various cancer cells, such as osteosarcoma and breast cancer cells, by inducing G1 cell cycle arrest and apoptosis (Liu et al., 2009). Coibamide A can reduce the drug resistance of tumors and prevent autophagic flux through the inhibition of autophagosome-lysosome fusion, thereby leading to caspase-independent death in tumor cells (Shi et al., 2021). It can also inhibit the migration, invasion, and cell cycle progression of glioblastoma and breast cancer cells (Hau et al., 2013). In addition, coibamide A possesses broad - spectrum activity, with a substrate overlap with apratoxin A, as exemplified by their shared targeting of HER/ErbB family proteins (Kazemi et al., 2021).

#### 4.2.4 Ipomoeassin F

Ipomoeassin F (Ipo-F) is a natural plant-derived resin glycoside cytotoxin that directly binds to Sec61α and exhibits potent anticancer activity in human breast cancer cells (MCF7) and lymphoma cells (U937) (Roboti et al., 2021; Zong et al., 2015). *In vitro* translocation assays showed that Ipo-F blocks all Sec61 substrates but does not inhibit insertion/translocation of tail-anchor, type III membrane proteins or short secretory proteins which can translocate independently of Sec61 (Zong et al., 2019). In addition to its anticancer efficacy, Ipo-F has been reported to exhibit antiviral activity by inhibiting the cotranslation of the SARS-CoV-2 spike protein and the host cell membrane receptor ACE2 (O'Keefe et al., 2021).

#### 4.2.5 Decatransin

Decatransin, a fungal-derived highly N-methylated cycloundecalactone peptide, exerts non-selective and broad-spectrum inhibitory effects on the translocation of polypeptides into the ER (Ohsawa et al., 2022). Studies have demonstrated that decatransin effectively suppresses the proliferation of cells by blocking Sec61-dependent protein translocation into the ER. This inhibitory mechanism operates independently of SRP-mediated recognition and SR-directed targeting processes, and is applicable to both co-translational and post-translational translocation pathways (Junne et al., 2015). Genetic studies have identified multiple decatransin-resistant mutations in Sec61α1, with the majority localized within the plug domain. Notably, the Q129L mutation in yeast Sec61α (orthologous to Q127L in human Sec61α) confers strong to moderate resistance against decatransin, suggesting that this residue plays a critical role in decatransin binding and function (Itskanov et al., 2023).

#### 4.2.6 Eeyarestatin

Eeyarestatin (ES), including eeyarestatin I (ESI) and eeyarestatin II (ESII), is an ERAD inhibitor (Cross et al., 2009). As the Sec61 translocon is closely linked to ERAD, inhibiting ER protein transfer may also block the retrotranslocation of misfolded proteins (Wang et al., 2010). In Alzheimer’s, Parkinson’s, prion, and Huntington’s diseases, protein degradation is impaired (Smith, 2018). ES inhibits ERAD, causing misfolded protein accumulation in the ER and inducing ER stress, offering a potential intervention strategy (Fiebiger et al., 2004). ES’s anticancer potential is also promising. ESI, like bortezomib, kills tumor cells by disrupting ER homeostasis and inducing ER stress. In NSCLC xenograft models, inhibiting valosin-containing proteins reduces tumor growth (Valle et al., 2011). Moreover, ESI combined with proteasome inhibitors like bortezomib shows enhanced antitumor effects. In antimicrobial research, ES24, an ES analog, inhibits SecYEG-dependent protein translocation and membrane insertion in *E. coli* (Steenhuis et al., 2021). Notably, ESI at 0.2–5 μM for 4 h eliminates the infectivity of Zika and Usutu viruses in a dose- and time-dependent manner, highlighting ES’s potential in antiviral therapy (Rodrigo et al., 2022). Overall, ES shows broad application potential in disease treatment.

## 5 Challenges and prospects

In this review, we have summarized the structure and function of the Sec61 protein and the role of SEC61 mutations in genetic diseases and cancer. As research on Sec61 continues to grow, Sec61 has become increasingly recognized as a therapeutic target for genetic diseases and cancers. However, the role of the Sec61 protein and its related proteins in the disease process is still unclear, and little is known about the cotranslational and posttranslational substrate proteins involved. With the development of high-throughput omics technology, we can screen substrates of the Sec61 complex and their modification methods via various methods. Currently, Sec61 inhibitors are still in the clinical trial stage as targeted therapies for cancer. Additional research is needed to determine whether the role of Sec61 in ER protein import and/or Ca^2+^ homeostasis is related to the observed clinical course of human cancers. Therefore, exploring the specific roles of the Sec61 complex in different tissues will play a crucial role in the therapeutic application of Sec61 inhibitors.
